# Motor–Cognitive Treadmill Training With Virtual Reality in Parkinson’s Disease: The Effect of Training Duration

**DOI:** 10.3389/fnagi.2021.753381

**Published:** 2022-01-05

**Authors:** Elisa Pelosin, Chiara Ponte, Martina Putzolu, Giovanna Lagravinese, Jeffrey M. Hausdorff, Alice Nieuwboer, Pieter Ginis, Lynn Rochester, Lisa Alcock, Bastiaan R. Bloem, Freek Nieuwhof, Andrea Cereatti, Ugo Della Croce, Anat Mirelman, Laura Avanzino

**Affiliations:** ^1^Department of Neuroscience, Rehabilitation, Ophthalmology, Genetics and Maternal Child Health, University of Genoa, Genova, Italy; ^2^IRCCS Ospedale Policlinico San Martino, Genova, Italy; ^3^Center for the Study of Movement, Cognition and Mobility, Neurological Institute, Tel Aviv Sourasky Medical Center, Tel Aviv, Israel; ^4^Sackler School of Medicine and Sagol School of Neuroscience, Tel Aviv University, Tel Aviv, Israel; ^5^Department of Physical Therapy, Tel Aviv University, Tel Aviv, Israel; ^6^Department of Orthopedic Surgery, Rush Alzheimer’s Disease Center, Rush University Medical Center, Chicago, IL, United States; ^7^Department of Rehabilitation Sciences, Neurorehabilitation Research Group (eNRGy), KU Leuven, Leuven, Belgium; ^8^Translational and Clinical Research Institute, Newcastle University, Newcastle upon Tyne, United Kingdom; ^9^Newcastle upon Tyne Hospitals NHS Foundation Trust, Newcastle upon Tyne, United Kingdom; ^10^Department of Neurology, Radboud University Medical Centre, Centre of Expertise for Parkinson and Movement Disorders, Donders Institute for Brain, Cognition and Behaviour, Nijmegen, Netherlands; ^11^Department of Biomedical Sciences, University of Sassari, Sassari, Italy; ^12^Department of Electronics and Telecommunications, Politecnico di Torino, Turin, Italy; ^13^Department of Experimental Medicine, Section of Human Physiology, University of Genoa, Genoa, Italy

**Keywords:** Parkinson’s disease, treadmill training (TT), virtual reality, gait, cognitive functions, falls

## Abstract

Treadmill training with virtual reality (TT + VR) has been shown to improve gait performance and to reduce fall risk in Parkinson’s disease (PD). However, there is no consensus on the optimal training duration. This study is a sub-study of the V-TIME randomized clinical trial (NCT01732653). In this study, we explored the effect of the duration of training based on the motor–cognitive interaction on motor and cognitive performance and on fall risk in subjects with PD. Patients in Hoehn and Yahr stages II–III, aged between 40 and 70 years, were included. In total, 96 patients with PD were assigned to 6 or 12 weeks of TT + VR intervention, and 77 patients completed the full protocol. Outcome measures for gait and cognitive performance were assessed at baseline, immediately after training, and at 1- and 6-month follow-up. The incident rate of falls in the 6-month pre-intervention was compared with that in the 6-month post-intervention. Dual-task gait performance (gait speed, gait speed variability and stride length under cognitive dual task and obstacle negotiation, and the leading foot clearance in obstacle negotiation) improved similarly in both groups with gains sustained at 6-month follow-up. A higher decrease in fall rate and fear of falling were observed in participants assigned to the 12-week intervention than the 6-week intervention. Improvements in cognitive functions (i.e., executive functions, visuospatial ability, and attention) were seen only in participants enrolled in 12-week training up to 1-month follow-up but vanished at the 6-month evaluation. Our results suggest that a longer TT + VR training leads to greater improvements in cognitive functions especially those directly addressed by the virtual environment.

## Introduction

There is increasing evidence on the impact of the motor and cognitive interaction on mobility in Parkinson’s disease (PD) (Williams-Gray et al., 2007; Karachi et al., 2010; Williams-Gray and Worth, 2016; Nieuwhof et al., 2017; Saga et al., 2017). With disease progression, motor and cognitive dysfunctions and specifically the interaction between them increase the risk of adverse mobility outcomes such as falls, especially during more complex daily routines (Fasano et al., 2017; Pelicioni et al., 2020; Sharon et al., 2020).

Following these observations, in recent years, great effort has been dedicated to developing rehabilitative strategies targeting motor–cognitive interactions to improve gait performance and reduce fall risk in PD (Nieuwboer et al., 2007; Rochester et al., 2010; Mirelman et al., 2016; Strouwen et al., 2017; Silva-Batista et al., 2018; Penko et al., 2019; Pazzaglia et al., 2020; Imbimbo et al., 2021). Overall, results show that training based on motor–cognitive approaches is effective in reducing falls (Mirelman et al., 2016; Penko et al., 2019) and fear of falling (Nieuwboer et al., 2007; Silva-Batista et al., 2018) and leads to sustained improvements in usual and complex gait [e.g., dual task (DT)] (Rochester et al., 2010; Mirelman et al., 2016; Strouwen et al., 2017; Pazzaglia et al., 2020; Imbimbo et al., 2021).

However, data on the impact of this training on cognitive function are still less. In fact, many of the abovementioned studies were designed to improve dual-task gait performance and to impact the number of falls, but the effect on cognitive functions has not been systematically addressed.

In aging, evidence related to the effect of combined motor and cognitive training on cognition shows that in order to obtain improvements in cognitive functions, a long period of training is needed (Joubert and Chainay, 2018). However, this has not been systematically explored in a neurodegenerative condition such as PD. Elucidating the optimal duration of motor–cognitive combined approaches in PD to impact both motor and cognitive functions is an unmet need, which is critical for providing the best possible care but also for allocating sufficient healthcare resources.

In this study, we explored the effect of the duration of training based on the motor–cognitive interaction on motor and cognitive performance and on fall risk in subjects with PD. Data from two subsets of patients with PD recruited through the V-TIME project (Mirelman et al., 2013, 2016), differing for training duration, were compared. In brief, the V-TIME training protocol consisted in combining treadmill training and virtual reality (TT + VR) environment in order to train gait under challenging conditions (Mirelman et al., 2016). Patients underwent either 6 weeks of TT + VR training (3 sessions/week) or 12 weeks of TT + VR training (3 sessions/week). We hypothesized that increasing the duration of TT + VR training would lead to greater improvements and longer-lasting effects in complex gait (i.e., gait speed during DT), cognitive abilities [i.e., executive functions (EFs)], and would further reduce falls [i.e., reduced fall incident rate (IR)].

## Materials and Methods

### Study Design

**FIGURE 1:**
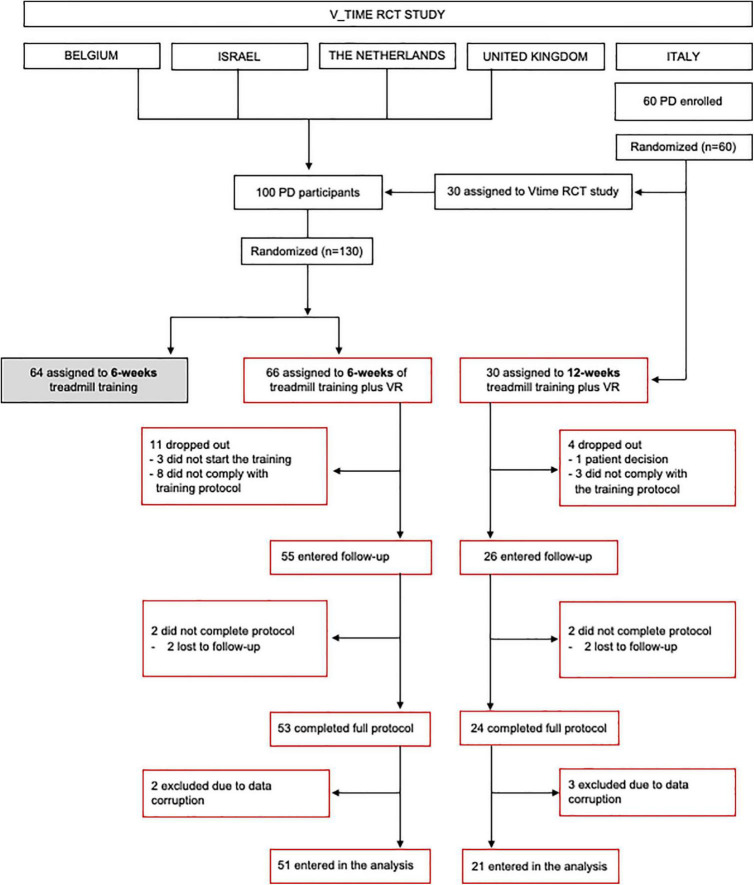
CONSORT diagram.

This is a preplanned sub-study of the randomized controlled V-TIME project (NCT01732653) (Mirelman et al., 2016) aimed at investigating the effect of training duration on the motor, cognitive, and fall risk in PD. Participants recruited in Italy were randomly assigned, following simple randomization procedures (computerized random numbers), to enter either the V-TIME RCT main protocol (6 weeks of TT or TT + VR training) or to receive 12 weeks of TT + VR training (Figure 1). Participants recruited at the other sites (i.e., Belgium, Israel, the Netherlands, and the United Kingdom) were randomly assigned to enter one of the two arms of the V-TIME RCT main protocol (6 weeks of TT or TT + VR training). In this sub-study, we included only patients assigned to TT + VR training (either 6-week or 12-week training). All participants were blinded to our hypothesis and expected outcomes and received the treatment by an unblinded trainer.

### Participants

Details of the recruitment of participants are provided in the flow diagram (Figure 1). Inclusion criteria were as follows: (1) diagnosis of idiopathic PD according to the United Kingdom Brain Bank criteria (Hughes et al., 1992), (2) 2 or more falls in the previous 6 months, (3) aged 60–85 years, (4) Hoehn and Yahr (H&Y) stage II or III (Hoehn and Yahr, 1967), (5) able to walk for 5 min unassisted, and (6) stable anti-Parkinsonian medication regimen for the past 1 month. Exclusion criteria were as follows: (1) past history of neurological conditions other than PD, (2) Mini-Mental State Examination (MMSE) (Folstein et al., 1983) score <21, (3) psychiatric co-morbidity (e.g., major depressive disorder as in accordance with DSM IV criteria), (4) unstable medical condition in the past 6 months, and (5) unable to comply with the training or currently participating in another trial. Age, gender, and years of education were recorded for all participants. Disease severity was assessed using the Movement Disorders Society Unified Parkinson’s Disease Rating Scale (MDS-UPDRS) (Goetz et al., 2008) and H&Y score. Informed written consent was obtained from all participants. This study was approved by local Ethics Committees (141/12) and registered in clinicalTrials.gov (NCT01732653). Additional details on V-TIME are published elsewhere (Mirelman et al., 2016).

### Intervention

Training was provided to both groups with the same TT + VR system (V-TIME). The system consisted of a camera (Kinect^®^), which is used to collect the feet movement of participants while walking on the treadmill and to incorporate it into a computer-generated stimulation, which was presented to participants over a screen placed in front of the treadmill (Mirelman et al., 2013, 2016). In brief, patients were required to walk on the treadmill while avoiding virtual obstacles projected on the screen. The virtual environment is comprised of enriched visual stimuli engaging several cognitive domains such as EF (e.g., decision-making and planning), attention (e.g., ignoring distractors on the way), working memory (e.g., navigation), and visual processing (e.g., timing of motor planned action).

Training progression was structured according to a prespecified plan and was adjusted to increase the difficulty across the sessions. The 6-week group underwent 18 sessions of TT + VR training (3 sessions/week for 6 weeks), and the 12-week group was trained for a longer period of time, 36 sessions (3 sessions/week for 12 weeks). Each session lasted approximately 45 min. All interventions were delivered by physiotherapists. Details of training progression for both groups are fully reported in Supplementary Tables 1, 2. Regarding the 6-week training, the challenges of the long-training period were to maintain motivation and participation throughout the training period and to gradually increase the endurance level of the participants to such long-duration training. As such, in the 12-week group, it has been decided to grade the training progression in a more moderate way to allow for building the necessary endurance and to investigate whether such a progression could perhaps enhance motor learning and provide better retention of training effects.

### Assessment Protocol

Clinical and instrumental evaluations were performed before (PRE), immediately after (POST), 1 month after (FU-1m) the training, and 6-month post-intervention (FU-6m) by a blinded assessor. Testing was carried out while patients were ON medication and at the same time of day for each subject.

Gait performance was measured in wide and well-lit laboratories where participants were asked to walk under the following three conditions each lasting 1 min: (1) walking at their preferred speed (usual walking, UW), (2) walking while performing a verbal fluency (DT), and (3) walking while negotiating physical obstacles (obstacle, OB). Spatio-temporal parameters were recorded by small, lightweight 3 axial accelerometers (APDM, OR, United States) placed on both feet and on the back (L5) of each participant during all gait measurements. Data were collected at 240 Hz and analyzed using customized software.

Cognitive functions were assessed using a computerized neuropsychological test battery (NeuroTrax Corp., Medina, Modiin, Israel) and clinical scales. The NeuroTrax*™* assesses multiple cognitive domains including attention, memory, and EF and consists of the following five tasks: (1) “Stroop test,” which captures response inhibition to incongruent stimuli, (2) “Go-NoGo,” which is a variant of the continuous performance test, (3) verbal and (4) non-verbal memory tasks, which measure immediate and delayed recall, and (5) a “Catch game,” which tests set-shifting, adaptation, and planning. An objective cognitive function profile is produced with a global cognitive score (GCS) and five individual domain scores [i.e., memory, EF, visuospatial (VS) ability, attention, and information processing speed].

Related to falls, we collected the fall rate for 6 months before and after training. Fall rate before training was collected retrospectively by asking the patients the number of falls in the previous 6 months. Fall rate during the 6 months after the end of the training was collected by means of a fall calendar provided either in a paper version, web-based calendar, or a smartphone application (Mirelman et al., 2016). Fear of falling was measured using the Falls Efficacy Scale-International (FES-I) (Michalska et al., 2020) at PRE, POST, FU-1m, and FU-6m visits.

### Outcome Measures

The primary gait outcome measure was gait speed under DT condition. Secondary outcomes included several gait variables related to fall risk: gait speed under UW and OB, gait speed variability, and stride length under all the conditions (UW, DT, and OB), as well as leading and trail feet clearance under OB (Bertoli et al., 2018).

For cognition, the primary outcome measure was the EF score. Secondary outcome measures included scores of the other domains within the NeuroTrax*™* battery.

For falls, the primary outcome was the IR of falls during the 6 months after the end of the training, and the secondary outcome was the FES-I score.

### Statistical Analyses

Descriptive statistics are reported as means ± SDs. Prior to the analysis, all variables were examined for normality (Shapiro–Wilk test). At the baseline, a Chi-square test was applied to assess gender differences between groups. Differences for age and education were assessed by the non-parametric Mann–Whitney test, whereas a *t*-test was used to compare other clinical data (H&Y, disease duration). The MDS-UPDRS, UW, DT, and OB gait parameters were subjected to an RM-ANOVA with GROUP (6 weeks and 12 weeks) as between subject factors and VISIT (PRE, POST, FU-1m, and FU-6m) as within subject factor. Missing data resulting from dropouts, technical problems, and human errors were not imputed. Changes from baseline for NeuroTrax GCS and memory, EF, VS ability, attention, and information processing speed domains were analyzed using a generalized estimating equation (GEE) model adjusted for education (years) at each testing time (PRE, POST, FU-1m, and FU-6m). The level of significance was set at *p* < 0.05, and *post hoc* analysis was performed using *t*-tests. For comparisons between groups, the IR of falls with 95% CIs was calculated using a negative binomial regression model. The training group was the fixed factor, and the number of days after training was an offset variable. Age and sex were inserted as covariates in all the models used for statistical analysis. The level of significance was set at 5%, and *post hoc* analysis was performed using *t*-tests. Subsequently, to account for multiple comparisons, we applied the false discovery rate (FDR) method. Statistical analysis was performed using SPSS Statistics Version 21 (IBM Corp., Armonk, NY, United States).

## Results

### Participants

The study flow diagram is presented in Figure 1. At the end of the study, 77 participants (6-week group: *n* = 53, 12-week group: *n* = 24) completed the full protocol, but data from five participants were not included in the analysis due to data corruption. There were no reported medication changes during the trial. Group demographics and clinical characteristics are presented in Table 1. At baseline, the two groups were similar in age, gender, disease duration, H&Y stage, MDS-UPDRS sub-scores, and MMSE score (*p* > 0.05). Statistical analysis revealed a slight difference between groups for years of education (*p* = 0.043).

**TABLE 1:** Demographic and clinical characteristics of PD subjects entered in the analysis.

Measures	Group		*P*-value
	6-Weeks	12-Weeks	
Age, y	73.84 ± 6.39 (60–86)	74.09 ± 4.96 (65–83)	0.872
Sex (F–M)	20–31	9–12	0.775
Education, y	12.33 ± 3.79 (5–19)	10.23 ± 4.21 (5–18)	0.043*
Disease duration, y	8.47 ± 4.95 (2–20)	7.43 ± 4.26 (2–19)	0.286
H&Y stage	2.44 ± 0.46 (2–3)	2.48 ± 0.43 (2–3)	0.768
MDS-UPDRS I	11.90 ± 4.63 (3–24)	9.95 ± 4.04 (1–21)	0.097
MDS-UPDRS II	16.41 ± 7.31 (2–38)	14.00 ± 4.33 (4–24)	0.163
MDS-UPDRS III	32.33 ± 14.65 (6–76)	30.66 ± 9.22 (16–55)	0.631
MDS-UPDRS IV	2.88 ± 3.94 (0–13)	3.28 ± 2.66 (0–8)	0.669
MMSE	27.52 ± 1.72 (24–30)	28.19 ± 1.25 (25–30)	0.116
MOCA	24.23 ± 2.71 (20–30)	25.47 ± 2.52 (21–29)	0.077
Falls-6 m	3.58 ± 2.01 (2–12)	3.14 ± 1.76 (2–7)	0.920

Values are presented as mean ± standard deviation and () range. Y, years; F, female; M, male; H&Y, Hoehn and Yahr; MDS-UPDRS, Movement Disorder Society Unified Parkinson’s Disease Rating Scale; MMSE, Mini-Mental State Examination; MOCA, Montreal Cognitive assessment; 6 m, 6 months before the baseline. Asterisks indicate statistically significant difference at baseline between groups (*p < 0.05).

### Effect of Training Duration on Gait Performance

Means ± SDs and full details of the statistical analysis of gait parameters are reported in Table 2. Significant results reported survived FDR correction. In all, statistical analysis neither reveal any significant time × group interaction nor main group effect (always *p* > 0.05) for all gait parameters considered. However, we found a significant effect of time for gait speed (task: UW, *p*-adj = 0.0003; DT, *p*-adj = 0.0003; and OB, *p*-adj = 0.005), gait speed variability (task: UW, *p*-adj = 0.027; DT, *p*-adj = 0.004; and OB, *p*-adj = 0.002), stride length (task: UW, *p*-adj = 0.002; DT, *p*-adj = 0.002; and OB, *p*-adj = 0.003), and leading foot clearance (*p*-adj = 0.033). *Post hoc* analysis revealed that improvements were maintained up to 6-month follow-up (always *p*-adj < 0.05). Finally, no significant changes were found for clearance data during obstacle crossing (always *p* > 0.05).

**TABLE 2:** Spatio-temporal parameters under usual and complex walking.

	6-Weeks	12-Weeks	Time *p*-adjust	*Post-hoc* analysis Time (*p*-adjust)	Group*	Time × Group Interaction*
Gait speed usual walking	m/s	m/s				
Pre	0.93 ± 0.20	0.92 ± 0.10	***p* = 0.0003**		*p* = 0.372	*p* = 0.174
Post	1.12 ± 0.19	1.03 ± 0.16		***Pre-post p* = 0.0009**		
FU-1 m	1.06 ± 0.21	1.01 ± 0.15		**Pre – FU-1 m *p* = 0.0005**		
FU-6 m	1.01 ± 0.22	1.00 ± 0.15		**Pre – FU-6 m *p* = 0.0015**		
Gait speed DT walking	m/s	m/s				
Pre	0.81 ± 0.19	0.86 ± 0.22	***p* = 0.0003**		*p* = 0.981	*p* = 0.108
Post	0.99 ± 0.18	0.94 ± 0.20		***Pre-post p* = 0.0009**		
FU-1 m	0.96 ± 0.21	0.94 ± 0.16		**Pre – FU-1 m *p* = 0.0005**		
FU-6 m	0.92 ± 0.21	0.93 ± 0.17		**Pre – FU-6 m *p* = 0.0013**		
Gait speed OB walking	m/s	m/s				
Pre	0.93 ± 0.17	0.92 ± 0.17	***p* = 0.0005**		*p* = 0.368	*p* = 0.116
Post	1.10 ± 0.22	1.00 ± 0.17		***Pre-post p* = 0.0012**		
FU-1 m	1.05 ± 0.20	0.99 ± 0.15		**Pre – FU-1 m *p* = 0.0006**		
FU-6 m	1.00 ± 0.22	0.98 ± 0.12		Pre – FU-6 m *p* = 0.008		
Gait speed CV usual walking	%	%				
Pre	0.12 ± 0.08	0.12 ± 0.07	***p* = 0.027**		*p* = 0.060	*p* = 0.119
Post	0.11 ± 0.07	0.07 ± 0.02		***Pre-post p* = 0.013**		
FU-1 m	0.11 ± 0.08	0.08 ± 0.03		**Pre – FU-1 m *p* = 0.036**		
FU-6 m	0.11 ± 0.05	0.09 ± 0.03		**Pre – FU-6 m *p* = 0.033**		
Gait speed CV DT walking	%	%				
Pre	0.14 ± 0.06	0.14 ± 0.04	***p* = 0.004**		*p* = 0.247	*p* = 0.810
Post	0.12 ± 0.09	0.11 ± 0.05		***Pre-post p* = 0.029**		
FU-1 m	0.12 ± 0.06	0.09 ± 0.03		**Pre – FU-1 m *p* = 0.0003**		
FU-6 m	0.12 ± 0.05	0.10 ± 0.05		**Pre – FU-6 m *p* = 0.0002**		
Gait speed CV OB walking	%	%				
Pre	0.18 ± 0.06	0.20 ± 0.05	***p* = 0.002**		*p* = 0.502	*p* = 0.130
Post	0.16 ± 0.06	0.15 ± 0.03		***Pre-post p* = 0.0004**		
FU-1 m	0.17 ± 0.10	0.15 ± 0.02		**Pre – FU-1 m *p* = 0.013**		
FU-6 m	0.18 ± 0.05	0.16 ± 0.03		**Pre – FU-6 m *p* = 0.003**		
Stride length usual walking	m	m				
Pre	0.99 ± 0.20	0.95 ± 0.15	***p* = 0.0002**		*p* = 0.881	*p* = 0.414
Post	1.16 ± 0.18	1.17 ± 0.13		***Pre-post p* = 0.0003**		
FU-1 m	1.13 ± 0.18	1.14 ± 0.14		**Pre – FU-1 m *p* = 0.0002**		
FU-6 m	1.14 ± 0.19	1.14 ± 0.15		**Pre – FU-6 m *p* = 0.0002**		
Stride length DT walking	m	m				
Pre	0.96 ± 0.17	0.94 ± 0.13	***p* = 0.0002**		*p* = 0.307	*p* = 0.641
Post	1.13 ± 0.19	1.06 ± 0.18		***Pre-post p* = 0.0002**		
FU-1 m	1.10 ± 0.23	1.06 ± 0.17		**Pre – FU-1 m *p* = 0.0002**		
FU-6 m	1.06 ± 0.20	1.01 ± 0.19		**Pre – FU-6 m *p* = 0.0011**		
Stride length OB walking	m	m				
Pre	1.03 ± 0.15	0.99 ± 0.30	***p* = 0.0003**		*p* = 0.136	*p* = 0.740
Post	1.21 ± 0.18	1.14 ± 0.14		***Pre-post p* = 0.0003**		
FU-1 m	1.20 ± 0.20	1.12 ± 0.12		**Pre – FU-1 m *p* = 0.0002**		
FU-6 m	1.14 ± 0.25	1.11 ± 0.13		**Pre – FU-6 m *p* = 0.002**		
Leading foot clearance OB	cm	cm				
Pre	27.20 ± 8.5	26.83 ± 4.9	***p* = 0.033**		*p* = 0.896	*p* = 0.761
Post	30.48 ± 9.2	28.91 ± 7.5		***Pre-post p* = 0.017**		
FU-1 m	28.87 ± 8.5	28.88 ± 4.4		**Pre – FU-1 m *p* = 0.038**		
FU-6 m	28.61 ± 7.6	28.74 ± 4.5		**Pre – FU-6 m *p* = 0.040**		
Trail foot clearance OB	cm	cm				
Pre	30.06 ± 9.6	27.24 ± 7.2	*p* = 0.42		*p* = 0.533	*p* = 0.65
Post	30.81 ± 8.8	29.40 ± 9.5		NA		
FU-1 m	28.01 ± 7.2	28.45 ± 7.4		NA		
FU-6 m	28.18 ± 8.2	27.94 ± 6.6		NA		

Values are presented as mean ± standard deviation. DT, cognitive dual task; OB, obstacle negotiation task; CV, coefficient of variability; m/s, meter per second; m, meters; Pre, before training; Post, immediately after training (6 weeks or 12 weeks); FU-1 m, 1 month follow-up; FU-6 m, 6 months follow-up; NA, Not Applicable. P-adj, p-values obtained after having applied FDR correction. *FDR correction was not applied because p values were not significant. Bold values indicate statistically significant differences.

### Effect of Training Duration on Cognitive Function

**FIGURE 2:**
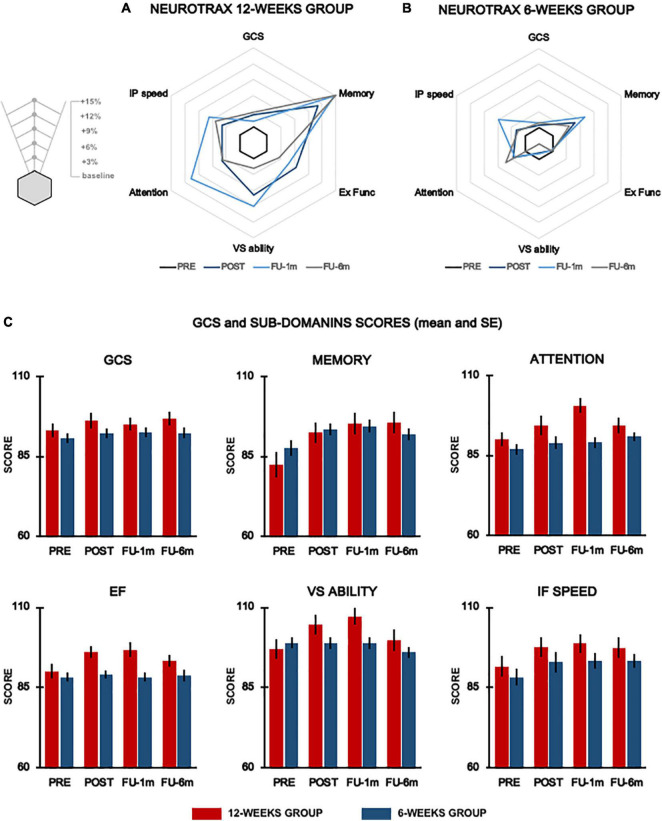
Cognitive function. Radar plots representing % changes from baseline of the global score and cognitive domains evaluated by means of NeuroTrax for the 12-week group **(A)** and 6-week group **(B)** at each testing time. Histograms show mean data for each group (red columns, 12-week group; blue columns, 6-week group) and vertical lines denote SE **(C)**. GCS, global cognitive score; Ex Func, executive functions; IP speed, information processing speed; VS ability, visuospatial ability.

The overall results of the comparison between the 6-week and 12-week groups for cognitive function measures are reported in Table 3 and represented in Figure 2. Before training, global and sub-domain scores from the computerized cognitive battery were similar between the two groups (GSC, *p* = 0.353; memory, *p* = 0.226; EF, *p* = 0.469; VS ability, *p* = 0.469; attention, *p* = 0.261; and information processing speed, *p* = 0.417).

**TABLE 3:** Neurotrax global and cognitive domains scores.

	6-Weeks	12-Weeks	Time *p*-adj	*Post-hoc* analysis *p*-adj	Time* Group *p*-adj	*Post-hoc* analysis *p*-adj	
Global cogntive score	Mean score ( ± SD)	Mean score ( ± SD)					
Pre	90.86 ± 10.95	93.19 ± 10.32	***p* = 0.0045**		*p* = 0.537	NA	
Post	92.22 ± 11.42	96.29 ± 10.82		**Pre-post *p* = 0.0003**			
FU-1 m	92.52 ± 10.68	95.16 ± 9.32		Pre – FU-1 m *p* = 0.18			
FU-6 m	92.35 ± 11.72	96.78 ± 9.95		Pre – FU-6 m *p* = 0.35			
Memory	Mean score ( ± SD)	Mean score ( ± SD)					
Pre	87.65 ± 18.16	82.35 ± 18.32	***p* = 0.0006**		*p* = 0.385	NA	
Post	93.33 ± 12.77	92.36 ± 15.19		***Pre-post p* = 0.0003**			
FU-1 m	94.48 ± 13.81	95.28 ± 15.29		**Pre – FU-1 m *p* = 0.0015**			
FU-6 m	91.94 ± 13.44	95.55 ± 15.64		**Pre – FU-6 m *p* = 0.0001**			
Executive functions	Mean score ( ± SD)	Mean score ( ± SD)					
Pre	88.30 ± 9.25	90.15 ± 10.45	***p* = 0.0084**		***p* = 0.0066**	**6-wk**	**12-wk**
Post	89.01 ± 8.63	96.79 ± 9.00		***Pre-post p* = 0.006**		*p* = 0.99	***p* = 0.036**
FU-1 m	88.92 ± 10.39	95.35 ± 10.23		**Pre – FU-1 m *p* = 0.0075**		*p* = 1.00	***p* = 0.036**
FU-6 m	89.16 ± 12.61	93.33 ± 12.61		Pre – FU-6 m *p* = 0.136		*p* = 1.00	*p* = 0.44
Visuo-spatial abilities	Mean score ( ± SD)	Mean score ( ± SD)					
Pre	99.01 ± 12.54	97.02 ± 13.69	***p* = 0.013**		*p* = 0.098†	NA	
Post	98.81 ± 13.14	104.66 ± 13.77		***Pre-post p* = 0.036**			
FU-1 m	99.00 ± 13.13	107.25 ± 11.58		*Pre-post p* = 0.071**†**			
FU-6 m	96.02 ± 15.21	99.76 ± 15.21		Pre – FU-6 m *p* = 0.843			
Attention	Mean score ( ± SD)	Mean score ( ± SD)					
Pre	86.95 ± 12.19	90.06 ± 10.06	***p* = 0.003**		*p* = 0.099†	NA	
Post	88.96 ± 14.27	96.44 ± 14.27		***Pre-post p* = 0.029**			
FU-1 m	89.10 ± 12.14	100.57 ± 10.40		**Pre – FU-1 m *p* = 0.003**			
FU-6 m	90.27 ± 10.79	94.28 ± 11.22		**Pre – FU-6 m *p* = 0.036**			
Info processing speed	Mean score ( ± SD)	Mean score ( ± SD)					
Pre	88.34 ± 18.96	91.70 ± 14.46	***p* = 0.004**		*p* = 0.957		
Post	92.97 ± 22.32	97.66 ± 14.45		***Pre-post p* = 0.003**			
FU-1 m	93.48 ± 17.53	98.81 ± 13.77		**Pre – FU-1 m *p* = 0.002**			
FU-6 m	93.46 ± 15.91	97.49 ± 15.01		**Pre – FU-6 m *p* = 0.002**			

*Values are presented as mean ± standard deviation (SD). 6-wk, 6-weeks training group; 12-wk, 12-weeks training group; Pre, before training; Post, immediately after training (6 weeks or 12 weeks); FU-1 m, 1 month follow-up; FU-6 m, 6 months follow-up; NA, Not Applicable; P-adj, p-values obtained after having applied FDR correction; †, p-values did not survive to FDR correction for multiple comparisons. Bold values indicate statistically significant differences.*

For EF, the primary outcome, we found a significant time × group interaction (*p*-adj = 0.066), and *post hoc* analysis revealed significant improvements (*p*-adj = 0.036) at post-evaluation only in the 12-week group. These improvements were maintained up to 1-month follow-up (*p*-adj = 0.036) but were not sustained at 6 month follow-up (*p*-adj = 0.44). We also found significant time × group interaction for VS ability (*p* = 0.049) and attention (*p* = 0.033) domains; however, these results did not survive FDR correction (VS ability, *p*-adj = 0.098; attention, *p*-adj = 0.099). Statistical analysis also revealed a significant effect of time for the EF (*p*-adj = 0.0084), GCS (*p*-adj = 0.0045), memory (*p*-adj = 0.0006), VS ability (*p*-adj = 0.013), attention (*p*-adj = 0.003), and information processing speed (*p*-adj = 0.004).

### Effect of Training Duration on Falls

**FIGURE 3:**
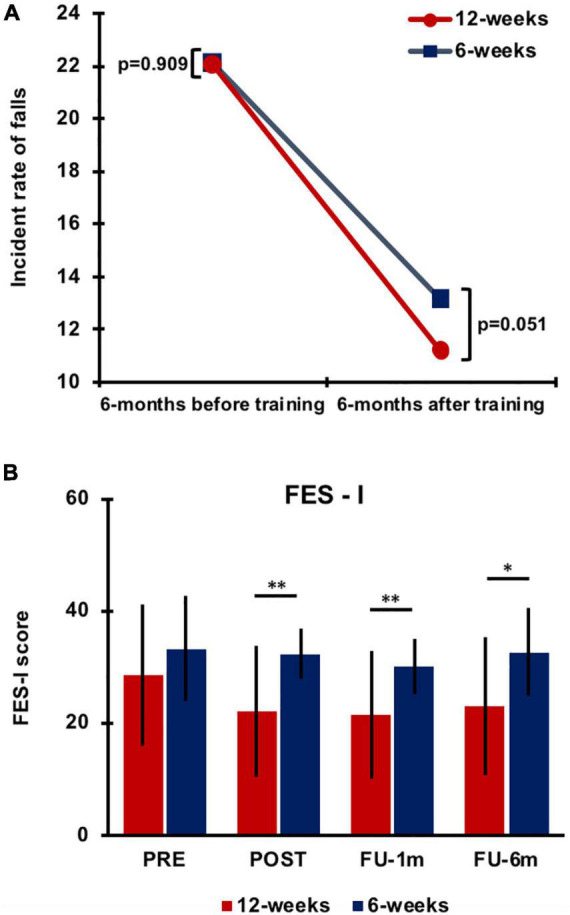
Differences in the incident rate of falls between training groups 6 months before and 6 months after training **(A)**. Falls Efficacy Scale-International (FES-I) score data (mean value) collected for the 6-week and 12-week groups at each testing time. Error bars indicate SD. Asterisks indicate the level of significance (**p < 0.001 and *p < 0.01) **(B)**. PRE, before training; POST, immediately after training; FU-1m, 1-month follow-up; and FU-6m, 6-month follow-up.

The overall results are presented in Figure 3. The fall IR 6 months before the intervention was similar between groups (6 weeks: IR: 22.13, 95% CI: 14.26–26.99; 12 weeks: IR: 22.05, 95% CI: 15.13–26.07; *p* = 0.909). At FU, the IR in the 6-week group was 13.12 falls (95% CI 9.82–18.64) with an improvement of 40.71%, while in the 12-week group, it was 11.17 ± 10.28 falls (95% CI 7.33–24.71) equal to 49.30% of improvement (Figure 3A). Overall, statistical analysis showed a significant change in the IR of falls over time (*p* = 0.003) for both groups with a strong trend for greater improvement in the 12-week group (*p* = 0.051). Notably, 63% of participants in the 6-week group and 75% in the 12-week group showed a reduction in fall rate compared to the self-reported fall rate prior to the study. Of note, 6% of participants in the 6-week group and 10% in the 12-week group showed no improvement after training in the number of falls compared to pretraining. In total, 31% of participants in the 6-week group and 15% in the 12-week group showed deterioration in the number of falls after training (i.e., more falls than pretraining).

A significant effect of time (*p* < 0.0001), group (*p* = 0.001), and time × group interaction (*p* = 0.048) was observed for fear of falling as evaluated with the FES-I. *Post hoc* analysis revealed improvements in both groups after training, but training-induced changes vanished after 1 month in the 6-week group, whereas they were retained up to 6-month follow-up in the 12-week group (Figure 3B; Supplementary Table 3). Significant differences were found between groups at each testing time after training (POST, *p* < 0.0001; FU-1m, *p* = 0.001; and FU-6m, *p* = 0.002).

## Discussion

In this study, we explored that if increasing the duration of a motor–cognitive VR training, it might induce additional benefits on gait performance, cognitive abilities, and falls in patients with PD. Our findings show that training duration acts differently on these domains. In fact, usual and complex (i.e., DT) gait performance improved after a short duration training, and training gains were maintained for 6 months with no further improvements by additional training sessions. Contrarily, cognitive functions required longer duration training to improve. In some cognitive domains, improvements were observed after the longer duration training only. Finally, the fall rate significantly improved already after the short duration training, however, the percent of patients who benefited from this treatment and incidence rate was slightly higher for patients in the 12-week training with a longer-lasting effect on fear of falling.

### Effect of Training Duration on Gait Performance

Contradictory to our hypothesis, the greater number of training sessions did not induce further improvements in gait performance. Significant improvements in gait speed (usual and DT) were observed and maintained for up to 6-month follow-up in both groups, regardless of the TT + VR training duration. Similar findings were also observed in the secondary outcome measures (gait speed variability under usual and DT and straight length and foot clearance under usual walking and obstacle negotiation), all improving significantly over time with no difference between groups.

In our experimental protocol, the progression of the “motor” component of the combined intervention (i.e., TT) was similar between the groups: gait speed increased gradually to 120% of individual walking speed in both training arms (Supplementary Tables 1, 2). Considering this, it is not surprising that improvements in usual walking performance were similar in the two groups. However, the exposure to the obstacles and cognitive load was twice as long in the long duration training as compared with the short duration training. As such, we expected that gait performance under cognitive DT and obstacle negotiation conditions would show greater improvements in the longer duration training group compared to the short duration training.

In accordance with our findings, a number of RCTs have shown that combined motor–cognitive interventions lead to long-lasting improvements on DT gait and balance performance (De Freitas et al., 2020) with a training duration of approximately 4–8 weeks (i.e., approximately 12–20 sessions). To our knowledge, this is the first study examining directly whether by doubling the number of training sessions, it is possible to impact more on dual-task gait performance. In this study, it appears that improvements under cognitive DT and obstacle negotiation improved quickly, already after 6 weeks of training, reaching a ceiling effect, and thus further improvements were not detected after the long-duration training. It is also possible that by improving the motor component in the DT, resources were vacant to deal with the cognitive component of the task.

### Effect of Training Duration on Cognitive Abilities

Our findings demonstrated that EFs benefited from the longer training duration in our cohort of subjects with PD. In fact, a significant improvement was observed only after the prolonged training (12 weeks). These results are in line with previous study in older adults which showed that combined physical and cognitive training leads to improvements in cognition (Colcombe and Kramer, 2003; Lauenroth et al., 2016). However, these studies stressed that several factors are crucial in influencing the efficacy of a combined physical and cognitive training on cognitive performance such as duration, frequency, and exercise modality. Related to training duration, a training scheme of 1–3 h weekly for 12–16 weeks (or more) has been suggested to be more likely to lead to detectable improvements in cognitive performance than shorter training schemes (Colcombe and Kramer, 2003; Lauenroth et al., 2016). Low-frequency and short-duration training (10 weeks, two sessions in a week), including gait training performed in single or DT, failed to show any significant change in cognition (San Martín Valenzuela et al., 2020). One such example is the SHARP-P trial, which failed to show significant improvements in an overall cognitive composite measure or in EF (Legault et al., 2011). In that study, the length of the training was appropriate (4 months) but the frequency of sessions was only twice a week with supervision. Moreover, the training protocol gradually “decreased” intensity setting a “low-challenges” schema for the cognitive training (Legault et al., 2011).

Our training design included a long duration trial (12 weeks, 36 sessions), and the training progression was based on increasing the cognitive challenges and the cognitive–motor interaction (obstacle level, modulation of distracters, and signposts) (Mirelman et al., 2013; Supplementary Material).

In addition, it is important to note that our VR training targeted especially the EFs that are necessary for accomplishing a training that mimics complex walking environments. There was also a trend toward larger improvements of attention and VS abilities after longer training with respect to the shorter one. In fact, these cognitive functions are also engaged by VR training.

Contrarily, memory and information processing speed were not explicitly addressed in the TT + VR training, which can explain the lack of additional improvements in these domains after the longer duration training. Finally, our results also show that the improvements observed in EFs were maintained up to 1 month after the end of the training. Noteworthy, at 6 months, FU performance on the EF test was similar to that observed at baseline. In the 12-week training program, this reflects an interval of 9 months of stable condition. Even if this is still a short time to drive clear conclusions, this finding is in line with a previous study suggesting that cognitive training may either briefly stabilize the cognitive decline in PD, delaying the downward trajectory or attenuate the rate of decline in PD (Walton et al., 2017).

The pathophysiology of cognitive deficits in PD is complex. Dopaminergic dysfunction (Bosboom et al., 2004; Caballol et al., 2007; Barone et al., 2011) as well as impairments in cholinergic (Bohnen et al., 2003, 2006; Hilker et al., 2005; Shimada et al., 2009) serotoninergic (Huot et al., 2011; Ye et al., 2014) and noradrenergic (Vazey and Aston-Jones, 2012; Del Tredici and Braak, 2013) pathways potentially contribute to cognitive deficits in PD. Previously, we demonstrated that training with TT + VR induced modulation of the pathological activation of the frontal cortex in patients with PD during motor imagery of a complex gait (Maidan et al., 2017) and an increased activity of cortical cholinergic circuits (Pelosin et al., 2020). TT + VR training might selectively affect acetylcholine release, allowing for normalization of prefrontal cortex activation resulting in improvements of EFs and attention that are under the control of cholinergic transmission and prefrontal cortex. Whether long duration training further potentiated these mechanisms remains to be explored.

### Effect of Training Duration on Falls

Our results showed a significant decrease in the IR of falls over time with a trend for greater improvement in the 12-week group. Furthermore, there was a slightly higher proportion of participants in the 12-week training who demonstrated a reduced number of falls compared with the period prior to the training (75%) and a much smaller percentage who deteriorated (15%), compared to those enrolled in the 6 weeks. Patients also reported less fear of falling after the 12-week training, and this gained confidence level was sustained for at least 6 months. The causes of falls in PD are multifactorial and extend beyond motor impairment. The severity of psychosis, executive cognitive impairment, autonomic (particularly cardiovascular) dysfunction, sleep disturbances, and polypharmacy have been associated with falls (Fasano et al., 2017; Giovannini et al., 2021). It has been suggested that patients with PD compensate for declined gait performance, by increasing reliance on cognition to control gait (Iansek et al., 2013). Falls may be the result of insufficient or ineffective compensation. Thus, some consider falls as a motor–cognitive failure or interference. Several theoretical models have been suggested to explain motor–cognitive interference (Strouwen et al., 2015). One such theory is the multiple resource model (Wickens, 2008), which proposes that resource competition occurs at multiple dimensions, thus successful multitasking depends on the capacity to simultaneously rely on multiple brain resources necessary to run the different components of the tasks. Based on this model, improvement in one component of the integrative motor–cognitive function may influence the interaction between these domains. Our findings show a significant reduction in falls already after 6 weeks of training (Mirelman et al., 2016) potentially mainly reflecting the effect of the motor improvements. The larger improvements in cognitive functions in the longer duration training in addition to motor performance improvement may be the underlying source of the larger proportion of patients with reduced falls and the additional reduction in fall incidence seen after 12 weeks of training.

Evidence from basic science supports the role of cholinergic hypo-function in PD inducing impairments in attention and thus increasing the risk of falls (Sarter et al., 2014). Sarter et al. (2014) proposed that when the loss of cortical cholinergic inputs impairs the attentional processing of gait, the striatal circuitry is “deprived” of this information, which it would normally use to select and sequence motor actions. In other words, dual cholinergic-dopaminergic loss attenuates the supervision of striatal circuitry and thereby “unmasks” the consequences of striatal dopaminergic denervation on gait and falls (Sarter et al., 2014). Following this hypothesis, it is possible that the 12-week training may have had a greater impact on falls by acting on the cognitive aspects of gait. Such findings suggest that a long duration TT + VR training may be especially beneficial to patients with mild cognitive impairments.

A relevant final aspect worthy to be considered concerning the decreased fall rate after TT + VR training is the one related to polypharmacy and the risk of falls in PD. A recent study showed that in elderly patients with PD, the number of daily medications is associated with an increased risk of hospitalization (Giovannini et al., 2021). In fact, approximately 28% of hospitalizations reported were due to bone fractures caused by fall episodes. The association between polypharmacy and risk of falls has also been highlighted in a previous study (Giovannini et al., 2018) of the same group in home care patients, particularly in relation to the assumption of some drugs (e.g., benzodiazepines) that are commonly used to treat non-motor symptoms in PD. It would be interesting to explore whether it is possible to reduce the number of medication (particularly those targeting non-motor symptoms in PD), influencing polypharmacy, and risk of falling, by applying a motor–cognitive training protocol, as the one adopted in this study.

Finally, it is important to consider that our results on IR of falls could have been influenced by a reporting bias, caused by retrospective recall used to estimate the number of falls over 6 months, before the training. In contrast, it has been shown that patients tend to underreport falls when asked to recall (Hoffman et al., 2018) and that a more objective recording results in more falls. This could support our findings showing a reduction in the number of falls 6-month post-intervention.

### Training Compliance

Participant dropouts during the training were similar between groups, approximately 12% in the 6-week group and 10% in the 12-week group (Chi-square: *p* = 0.68), suggesting that the duration of training did not affect the participation of patients. In all, 11 patients (8 in the 6-week and 3 in the 12-week groups) abandoned the training due to personal reasons not related to training compliance. Finally, none of the participants sustained severe adverse events during the study.

### Study Limitations

The current results have to be interpreted against the limitations of this study. First, the sample size of the 12-week group was smaller than that of the 6-week group, but this was taken into account in the statistical analysis. Second, we did not include an active control group for the 12-week training. However, the main V-TIME study included a randomized design with an active control group receiving only TT, showing significantly greater effects in the TT + VR on IR of falls.

Third, this study is a preplanned sub-study of the randomized controlled V-TIME project (Mirelman et al., 2016) in which the decision to exclude people with a clinical diagnosis of dementia or severe cognitive impairment was established in order to have a homogeneous sample among the three populations (i.e., idiopathic fallers, people with PD, and people with mild cognitive impairment) involved in the project. Nevertheless, given the particular beneficial effect of the longer training on cognitive outcomes, it would be interesting in future studies to test whether this type of training may be also beneficial for patients with more severe cognitive decline. Fourth, fall number 6 months before training was based on a self-reported estimate for which introduces well-known recall bias (Ganz et al., 2005). Fifth, the effects of training were only explored over a period of 6 months, and information on retention effects beyond this period is unavailable. Finally, the training was provided in a clinical setting under a strict protocol of three sessions per week. Such a protocol is beyond the standard of care in most countries. It is unknown if a more flexible intensity protocol or setting (i.e., training at home) will induce similar effects. This should be explored in future studies.

## Conclusion

Our results show that prolonged TT + VR training was more beneficial in improving cognitive functions, especially EFs, that are directly addressed by the virtual environment than a shorter duration training (6 weeks). Longer duration training may be especially beneficial for patients with mild cognitive impairment. Our findings open the door to tailored personalized treatments based on the motor and cognitive profiles of patients.

## Data Availability Statement

The raw data supporting the conclusions of this article will be made available by the authors, without undue reservation.

## Ethics Statement

The studies involving human participants were reviewed and approved by the Ethics Committee Regione Liguria. The patients/participants provided their written informed consent to participate in this study.

## Author Contributions

EP, AM, and LAv: data analysis, writing, and finalizing the manuscript. CP, MP, GL, PG, LAl, and FN: data collection and data analysis. AC and UD: data analysis. JH, AN, LR, and BB: data analysis and revising the manuscript. All authors contributed to this study and approved the submitted version.

## Conflict of Interest

The authors declare that the research was conducted in the absence of any commercial or financial relationships that could be construed as a potential conflict of interest.

## Publisher’s Note

All claims expressed in this article are solely those of the authors and do not necessarily represent those of their affiliated organizations, or those of the publisher, the editors and the reviewers. Any product that may be evaluated in this article, or claim that may be made by its manufacturer, is not guaranteed or endorsed by the publisher.
