# Supplementary material for: Motor–Cognitive Treadmill Training With Virtual Reality in Parkinson’s Disease: The Effect of Training Duration
Source: Front Aging Neurosci. 2022 Jan 5;13:753381. doi: 10.3389/fnagi.2021.753381 (PMC8767105; doi:10.3389/fnagi.2021.753381)
Supplement: Supplementary file 1 [file Table_1.docx]

**Table S1: Progression milestones per session and setting ranges in the 6-weeks TT+VR training group**

| **WEEKS** | **#** | **SPEED** | **ENVIRONMENT** | **DURATION**  **(minutes)** | **OBSTACLE LEVEL** | **VISIBILITY** | **SIGNPOSTS**  **LEVEL** | **DISTRACTERS** |
| --- | --- | --- | --- | --- | --- | --- | --- | --- |
| 1 | 1 | **80%** | **PARK** | 15 | Very Easy | Day | Easy | None |
|  | 2 |  |  |  |  |  |  |  |
|  | 3 |  |  | 15-20 |  |  |  |  |
| 2 | 4 | **90%** |  |  | Easy |  |  |  |
|  | 5 |  |  | 20-25 |  |  |  |  |
|  | 6 |  |  |  |  | Night |  |  |
| 3 | 7 | **100%** |  | 25-30 | Medium |  | Easy-Medium | Easy |
|  | 8 |  |  |  |  | Add low fog | Medium |  |
|  | 9 |  |  |  |  |  |  | Easy-Medium |
| 4 | 10 | **110%** |  | 30-35 | Hard |  |  |  |
|  | 11 |  | **PARK + URBAN** |  |  | Add med fog |  | Medium |
|  | 12 |  |  |  |  |  | Medium-Hard |  |
| 5 | 13 | **120%** |  | 35-40 | Very-Hard |  | Hard | Medium-Hard |
|  | 14 |  |  |  |  | Add high fog |  | Hard |
|  | 15 |  |  | 40-45 |  |  |  |  |
| 6 | 16 |  |  |  | Insane |  |  | Hard + Question |
|  | 17 |  |  |  |  | Diverse |  |  |
|  | 18 |  |  |  |  |  |  |  |
