# Supplementary material for: Motor–Cognitive Treadmill Training With Virtual Reality in Parkinson’s Disease: The Effect of Training Duration
Source: Front Aging Neurosci. 2022 Jan 5;13:753381. doi: 10.3389/fnagi.2021.753381 (PMC8767105; doi:10.3389/fnagi.2021.753381)
Supplement: Supplementary file 2 [file Table_2.docx]

**Table S2: Progression milestones per session and setting ranges in the 12-weeks TT+VR training group**

| **WEEKS** | **#** | **SPEED** | **ENVIRONMENT** | **DURATION**  **(minutes)** | **OBSTACLE LEVEL** | **VISIBILITY** | **SIGNPOSTS**  **LEVEL** | **DISTRACTERS** |
| --- | --- | --- | --- | --- | --- | --- | --- | --- |
| 1 | 1 | **80%** | PARK |  | Very Easy | Daytime | Easy | None |
|  | 2 |  |  | 15 |  |  |  |  |
|  | 3 |  |  |  |  |  |  |  |
| 2 | 4 |  |  |  |  | Daytime  Twilight  Night |  |  |
|  | 5 |  |  | 20 |  |  |  |  |
|  | 6 |  |  |  | Easy |  |  |  |
| 3 | 7 | **90%** |  |  |  |  |  |  |
|  | 8 |  |  | 25 |  |  |  |  |
|  | 9 |  |  |  |  |  |  |  |
| 4 | 10 |  |  |  |  |  | Medium | Easy-Medium |
|  | 11 |  |  | 30 |  |  |  |  |
|  | 12 |  |  |  | Medium |  |  |  |
| 5 | 13 | **100%** |  |  |  |  |  |  |
|  | 14 |  |  | 35 |  |  |  |  |
|  | 15 |  |  |  |  | Add Low Fog |  |  |
| 6 | 16 |  |  |  |  |  |  |  |
|  | 17 |  |  | 40 | Hard |  |  |  |
|  | 18 |  |  |  |  |  |  |  |
| 7 | 19 | **110%** |  |  |  | Add Medium Fog | Hard Park  Easy Urban | Hard |
|  | 20 |  |  | 45 |  |  |  |  |
|  | 21 |  |  |  |  |  |  |  |
| 8 | 22 |  |  |  |  | Add High Fog |  | Add Question |
|  | 23 |  |  | 45 |  |  |  |  |
|  | 24 |  | PARK + URBAN |  | Hard  Very Hard  (Park)  Easy-Medium  (Urban) | Diverse (Park)  Add low Fog  (Urban) | Hard  (Park)  Easy -  Medium  (Urban) | Hard  (Park)  None  (Urban) |
| 9 | 25 |  |  |  |  |  |  |  |
|  | 26 |  |  | 45-50 |  |  |  |  |
|  | 27 |  |  |  |  |  |  |  |
| 10 | 28 | **120%** |  |  |  | Diverse (Park)  Add medium Fog  Diverse + Add Fog  Low-High  Fog |  | Hard  (Park)  Easy -Medium  (Urban) |
|  | 29 |  |  | 45-50 |  |  |  |  |
|  | 30 |  |  |  |  |  |  |  |
| 11 | 31 |  |  |  | Hard  Very Hard |  | Hard |  |
|  | 32 |  |  | 45-50 |  |  |  |  |
|  | 33 |  |  |  |  |  |  | Hard  (Park + Urban) |
| 12 | 34 |  |  |  | Insane |  |  |  |
|  | 35 |  |  | 45-50 |  |  |  | Add  Questions |
|  | 36 |  |  |  |  |  |  |  |
