# Supplementary material for: Motor–Cognitive Treadmill Training With Virtual Reality in Parkinson’s Disease: The Effect of Training Duration
Source: Front Aging Neurosci. 2022 Jan 5;13:753381. doi: 10.3389/fnagi.2021.753381 (PMC8767105; doi:10.3389/fnagi.2021.753381)
Supplement: Supplementary file 3 [file Table_3.docx]

**Table S3.** Fall Efficacy Scale International (FES-I)

|  | **6-WEEKS** | **12-WEEKS** | **TIME** | **POST-HOC ANALYSIS**  **TIME** | **GROUP** | **TIME x GROUP**  **INTERACTION** | **POST-HOC ANALYSIS**  **GROUP** | **POST-HOC ANALYSIS**  **TIME** | |  |
| --- | --- | --- | --- | --- | --- | --- | --- | --- | --- | --- |
| **FES** | **score** | **score** |  |  |  |  | **6-WEEKS vs 12 WEEKS** |  | |  |
| Pre | 33.24±12.58 | 28.57±9.40 | **p<0.0001** |  | **p=0.001** | **p=0.048** | Pre – p=0,131 | **6-WEEKS** | **12-WEEKS** | |
| Post | 32.30±11.65 | 22.047±4.49 |  | **Pre – Post p=0.002** |  |  | **Post – p<0.001** | **p=0.045** | **p=0.001** | |
| FU-1m | 30.14±11.31 | 21.52± 4.98 |  | **Pre – FU-1m p<0.001** |  |  | **FU-1m – p=0.001** | **p=0.007** | **P<0.001** | |
| FU-6m | 32.64±12.36 | 23.00±7.83 |  | **Pre – FU-6m p=0.024** |  |  | **FU-6m – p=0.002** | p=0.682 | **p=0.016** | |
| Data are mean ± standard deviation. Pre, before training; Post, immediately after training (6 weeks or 12 weeks); FU-1m, 1 month follow-up; FU-6m, 6 months follow-up; NA, Not Applicable. | | | | | | | | | |  |
